# Annotations

**Published:** 1907-03-23

**Authors:** 


### ANNOTATIONS.

### Lord Lister's Eightieth Birthday.

ON April 5 will fall the eightieth anniversary of Lord Lister's birthday, and very naturally the suggestion has arisen that some special plan should be arranged adequately to mark the event. At the present day all opposition to, and criticism of, Lord Lister's work have disappeared under the influence of facts so numerous and so widespread as to leave no possibility of misinterpretation. Yet at the outset a very different atmosphere prevailed, and it is well both in justice to the individual and in the interests of the younger generation of practitioners that an attempt should be made to depict the change which has come over the whole of the surgical art as a result of the observations and experiments pursued by Lister in his early days as a surgeon to the Glasgow Royal Infirmary. Under modern conditions surgery conducts its undertakings with an ease and a confidence which seem part of the natural order of events. How far these results are the outcome of Lister's work can only be appreciated by those whose memories carry them back to the pre-antiseptic days and to the horrors of pyæmia and hospital gangrene. In view of all this, nothing can be more happy than the suggestion that the forthcoming anniversary should be celebrated by the issue of a volume containing Lord Lister's published scientific papers, preceded by a short biographical account of his life and work. Anyone wishing to take part in the tribute should communicate with Dr. C. J. Martin at the Lister Institute.

### Some Hysterical Neuroses of the Stomach.

IN an article on this subject Dr. Jas. Hendrie Lloyd draws attention to the curious habit known as *Merycism*, or chewing the cud. This *ruminatis humana* has been for the most part associated with various forms of mental or nervous disease, and has been recorded in several idiots and epileptics. Examples of this condition have been noted by early writers on medical subjects. Thus, in 1678, Ludovici wrote a work on the subject showing how the habit was occasionally observed in man and was identical with the condition seen in some of the lower animals. Peyer, in 1685, described twelve examples, and suggested that in such cases there existed a dilatation of the œsophagus constituting an *antrum cardiacum* or ante-stomach. Other authors have regarded the condition as a sign of hypochondriasis. A distinguished physiologist is reported to have acquired the habit as a result of self-experiment. Probably some of the cases recorded have been examples of what are now known as pharyngeal pouches, but there is no doubt that occasionally cases of true merycism are observed. There is actual regurgitation of food from the stomach and the patient chews it again, and appears to find some satisfaction or enjoyment in the act. Probably the act is at first volitional, but after a time becomes automatic, and thus may be compared with the habit spasms or *tics*. Another curious neurosis associated with the stomach is that of air-swallowing, the patient distending his stomach in this way with air, and then belching it up again. Here once more we seem to be near to the practice of swallowing numerous solid bodies which is seen not infrequently among the insane.

### Professional Dangers.

THE death of Dr. David Lowson calls renewed attention to the perils incident to the professional work of every medical man, for he died of blood-poisoning while operating three or four months ago on a case of appendicitis. Dr. Lowson was a graduate of the University of Aberdeen, who practised at Hull, where he filled the important post of surgeon to the Royal Infirmary. Whilst he was in practice at Huddersfield he was called upon to perform the operation of tracheotomy on a case of diphtheria. Whilst the operation was in progress the trachea became blocked with false membrane and blood. Dr. Lowson cleared it by suction and saved the life of his patient, though he infected himself with diphtheria and was incapacitated from work for a year. In recognition of this action he was awarded an Albert medal of the first class. Many valuable lives have been lost by the practice of sucking a tracheotomy wound in similar cases of emergency. But there can hardly be any case in which a surgeon is justified in resorting to such a dangerous method, which is almost certain to infect him with a very serious disease. The immediate dangers of a tracheotomy always seem greater than they are in reality. Even when the patient has ceased to breathe during the operation the opening of the trachea with all convenient speed and the subsequent performance of artificial respiration without attempting to introduce a tube, but merely with the edges of the trachea held apart, nearly always restore the patient to life. Blood can be prevented from entering the trachea by making the opening in it sufficiently large and bringing it up to the edges of the skin incision at once. Blockage of the tube by membrane is more difficult to treat, but it is generally sufficient to remove the membrane with a feather and to pick it out with a pair of laryngeal forceps. Poisoned wounds are an ever-present source of trouble to the medical profession, but they are rarely dangerous to those who are not overworked or depressed by some constitutional disturbance. Modern habits of more numerous holidays and greater exercise in the open air have reduced such wounds very greatly, for they were certainly much more frequent at a time when members of the profession frequented bars and billiard-rooms, and when all the wounds they dressed were suppurating. Syphilis contracted in the practice of their profession as accoucheurs is still of no uncommon occurrence, though the risk of inoculation can be reduced to a minimum by the wearing of rubber gloves in all doubtful cases, and by the careful disinfection of the abraded point where the surgeon only subsequently discovers the risk he has undergone. Recent work at the Institut Pasteur in Paris shows that it takes an appreciable time for syphilis to enter the system, and that if a mercurial ointment be rubbed into the seat of inoculation within an hour it will almost certainly destroy the infection, whilst if it be applied as late as 18 hours afterwards it may possibly arrest the transmission of the disease.

